# COVID-19 impact: Customised economic stimulus package recommender system using machine learning techniques

**DOI:** 10.12688/f1000research.72976.1

**Published:** 2021-09-16

**Authors:** Rathimala Kannan, Ivan Zhi Wei Wang, Hway Boon Ong, Kannan Ramakrishnan, Andry Alamsyah

**Affiliations:** 1 Department of Information Technology, Faculty of Management, Multimedia University, Cyberjaya, Selangor, 63100, Malaysia; 2 Faculty of Management, Multimedia University, Cyberjaya, Selangor, 63100, Malaysia; 3 Department of Economics, Faculty of Management, Multimedia University, Cyberjaya, Selangor, 63100, Malaysia; 4 Faculty of Computing and Informatics, Multimedia University, Cyberjaya, Selangor, 63100, Malaysia; 5 School of Economics and Business, Telkom University, Bandung, West Java, 40257, Indonesia

**Keywords:** COVID-19, low-income households, economic stimulus package, customisation, data analytics, machine learning, Gradient Boosted Tree

## Abstract

**Background**: The Malaysian government reacted to the pandemic’s economic effect with the Prihatin Rakyat Economic Stimulus Package (ESP) to cushion the novel coronavirus 2019 (COVID-19) impact on households. The ESP consists of cash assistance, utility discount, moratorium, Employee Provident Fund (EPF) cash withdrawals, credit guarantee scheme and wage subsidies. A survey carried out by the Department of Statistics Malaysia (DOSM) shows that households prefer different types of financial assistance. These preferences forge the need to effectively customise ESPs to manage the economic burden among low-income households. In this study, a recommender system for such ESPs was designed by leveraging data analytics and machine learning techniques.

**Methods**: This study used a dataset from DOSM titled “Effects of COVID-19 on the Economy and Individual - Round 2,” collected from April 10 to April 24, 2020. Cross-Industry Standard Process for Data Mining was followed to develop machine learning models to classify ESP receivers according to their preferred subsidies types. Four machine learning techniques—Decision Tree, Gradient Boosted Tree, Random Forest and Naïve Bayes—were used to build the predictive models for each moratorium, utility discount and EPF and Private Remuneration Scheme (PRS) cash withdrawals subsidies. The best predictive model was selected based on F-score metrics.

**Results**: Among the four machine learning techniques, Gradient Boosted Tree outperformed the rest. This technique predicted the following: moratorium preferences with 93.8% sensitivity, 82.1% precision and 87.6% F-score; utilities discount with 86% sensitivity, 82.1% precision and 84% F-score; and EPF and PRS with 83.6% sensitivity, 81.2% precision and 82.4% F-score. Households that prefer moratorium subsidies did not favour other financial aids except for cash assistance.

**Conclusion**: Findings present machine learning models that can predict individual household preferences from ESP. These models can be used to design customised ESPs that can effectively manage the financial burden of low-income households.

## Introduction

The novel coronavirus 2019 (COVID-19) pandemic has created devastation in people’s lives worldwide, both socially and economically ( Shah *et al.*, 2020). As a result, governments have adopted various strategies aimed at reducing the pandemic’s impact, particularly the financial strain. The Malaysian government has introduced a series of economic stimulus packages to support various segments of its citizens. One such support is the Prihatin Rakyat Economic Stimulus Package (ESP) to cushion the impact of COVID-19 on low-income households after the first movement control in the country. The ESP consists of cash assistance, utility discount, moratorium, Employee Provident Fund and Private Remuneration Scheme (EPF and PRS) cash withdrawals and Credit Guarantee Scheme and Wage subsidies ( Flanders *et al.*, 2020). Following the implementation of ESP, the Department of Statistics Malaysia (DOSM) carried out a special survey from April 10 to April 24,2020 to better understand the implications of COVID-19 on the economy and households. The study included questions on social and economic factors and subsidy preferences.

A typical low-income household often bears considerable debt and has limited savings. When movement control was implemented, households that lost their income sources faced difficulties in accessing necessities, such as food and housing ( Flanders *et al.*, 2020). Even though the government offered ESP to help residents cope financially, the demands and desires of citizens in the event of a pandemic are unknown. For example, several households are reluctant to withdraw from EPF and PRS due to its reduction on their savings for old age. A personalised ESP can be built to reduce residents’ financial burden in this crisis if we can foresee their requirements and preferences for various subsidies, such as cash allowance, utility discount, moratorium or EPF and PRS withdrawals. Using data analytics and machine learning approaches, this study attempted to analyse survey data and construct predictive models for customised economic stimulus packages. The following research questions were put forward.

1. Can we develop a general profile of households who prefer moratorium subsidies?

2. Can we develop a general profile of households who prefer utility discount subsidies?

3. Can we develop a general profile of households who prefer EPF and PRS withdrawals?

This study contributes to the literature by using four machine learning techniques on socioeconomic survey data and predicting household subsidy preferences. A comparison of the feature selection methods, such as Gini index, Gain–Ratio and various partitioning ratios of the training and test data sets were carried out. The outcomes of this study can help the government deliver better and improved stimulus packages in the future based on individual preferences.

## Methods

**Figure 1.**
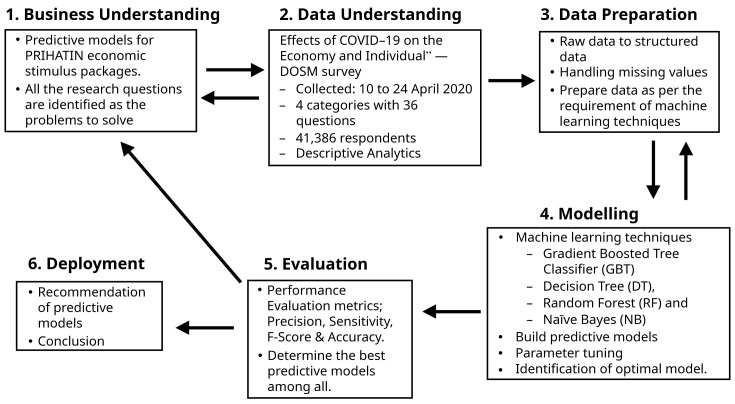
Research methods.

For planning and execution, this study used the Cross-Industry Standard Process-Data Mining (CRISP-DM), which is the industry-independent *de-facto* standard for implementing data mining initiatives ( Schröer *et al.*, 2021). This process has six phases, namely, business understanding, data understanding, data preparation, modelling, evaluation and deployment. Figure 1 depicts the activities carried out in each phase, as further explained below. 

### Business understanding

This phase identifies the problems to solve using the machine learning perspective and approach. All three research questions were selected as the problems, and the purpose was to propose predictive models for the moratorium, utility discount and EPF and PRS subsidies in the Prihatin Rakyat ESPs.

### Data understanding

Data gathering, evaluating, characterising and assuring its quality are part of this phase. DOSM performed a special survey (Round 2) to investigate the consequences of the COVID-19 epidemic on household economics and status ( “Department of Statistics Malaysia Official Portal,” 2020; Malaysia, 2020). The dataset includes 36 questions and 41,386 respondents. However, the data obtained from DOSM were not complete due to missing questions. The missing questions were Q3, Q6, Q19, and Q27 – Q31. In terms of the total respondents, the data were complete and had a total of 41,386 participants, all of them were aged 15 and older. 96.8% of the respondents have received benefits from Prihatin Rakyat ESPs. The raw data were based on responses from respondents, which included qualitative personal opinions on economy, employment, lifestyle, and education. The original dataset was in Malay language, and is translated into English for this study and given below as Table 1.

**Table 1.** Survey questions.

1. Joined and answered Survey Round 1 (Yes, No) 2. State (KL, Johor, Selangor, Perak, Sarawak, Kedah, Pahang, Putrajaya, Perlis, Kelantan, Melaka, Pulau Pinang, Labuan, Terengganu, Negeri Sembilan, Sabah) 3. 4. Gender (Female, Male) 5. Stay in (City, Rural) 6. 7. Age group (15–24, 25–34, 35–44, 45–54, 55–64, 65 and above) 8. Marital status 9. (Single/Non-married, Married, Single mother, Widow/Widower, Divorced/Separated) 10. Number of dependents (including respondents) (1–2, 3–4, 5–10, More than 10 people) 11. Ethnic groups (Malay, Indian, Native Sabah/Sarawak, Chinese, Others, Foreigner) 12. Benefit from the PRIHATIN Economic Stimulus Package (ESP)? (Yes, No) 13. The most advantageous form of assistance received under the PRIHATIN Economic Stimulus Package? a. Cash assistance (National Prihatian assistance, IPT student assistance, E-hailing) (Yes, No) b. Utilities discount (Yes, No) c. Moratorium (Yes, No) d. EPF cash withdrawals & Private remuneration scheme (Yes, No) e. Credit guarantee scheme (Yes, No) f. Wage subsidies and payments under ERP program (Yes, No) g. NA (Yes, No) 13. Process/procedure achievement level for PRIHATIN assistance (Easy, Difficult, Medium, NA, Others) 14. Satisfied with the help of PRIHATIN (Yes, No, NA) 15. Status of eligibility as a recipient of PRIHATIN aid (Eligible, New application, Appeal, Not eligible) 16. Ranking according to your preferences with the situation facing now [Health & lives] (1,2,3) a. Ranking according to your preferences with the current situation [Income (employment/ business/enterprise)] (1,2,3) b. Ranking according to your preferences with the situation faced now [Life goes back as normal] (1,2,3) 17. The impact of the PRIHATIN Economic Stimulus Package (Effective, Most effective, Others, NA, No effects) 18. With the extension of the Movement Control Order (MCO) until 28 April 2020, does it still require assistance/ the PRIHATIN Economic Stimulus Package for the next phase? (Yes, No) 19. 20. Views on health workers (frontlines) and health facilities provided by the government in dealing with COVID-19 (Good, Bad) 21. Views on achievements and facilities provided in dealing with COVID-19 in Malaysia compared to other countries. (Good, Bad) 22. COVID-19 outbreak affects lifestyle (Yes, No) 23. Ready to have a new normal lifestyle (i.e. a new normal life will differ from normal life before the spread of the COVID-19 outbreak) (Yes, No) 24. If ready, lifestyle changes will be done a. Number of lifestyle changes to be done (Yes, No) b. Will not eat out (Yes, No) c. Limiting social activities (Yes, No) d. Limiting sports and recreational activities (Yes, No) e. Limited religious and spiritual activity at home (Yes, No) f. Limiting tourism activities (Yes, No) g. Improves hygiene (Yes, No) h. Others (Yes, No) i. NA (Yes, No) 25. Working during the MCO period (Work from home, Not working, Rotation - partial payment, Full paid leave, Rotation - full payment, Semi-salary leave, Retrenched) 26. Work as (Government servants, Private sector employees, Not working, Self-employed, GLC employees, MNC employees, employers, Unpaid family workers) 27. 28. 29. 30. 31.	32. Main expenses on food products during MCO (Yes, No) a. Number of major food products expenditure during MCO b. Dry food items (e.g. bihun, biscuits, nestum, etc.) c. Cooked food (takeaway/delivery) d. Instant noodles e. Eggs f. Fish/chicken/meat/seafood for cooking g. Vegetables h. Fruits i. Frozen food (e.g. sausages, nuggets, fish balls, french fries, etc.) j. Rice k. Spaghetti l. Bread m. Baby food n. Animal food o. Cooking oil p. Essential items for cooking (example: shallots, garlic, sugar, salt, etc.) q. Canned beverages (examples: milo, condensed milk, powdered milk, etc.) r. Flour (including wheat flour, Rice flour, glutinous flour, etc.) s. 3-in-1 Drinks t. Vitamins/Supplements u. Drinking water v. Othersr 33. Main expenses for non-food products during MCO (Yes, No) a. Number of main expenses on non-food during MCO b. Hand wash c. Tissue paper d. Disinfectant e. Face mask f. Baby diaper g. Wet tissue h. Sanitary pad i. Laundry soap j. Toiletries k. Thermometer i. Medications (e.g. fever medicine, flu medicine, cough medicine, etc.) m. First aid kit n. Gloves (various sizes) o. Others 34. Internet access level for yourself / your child's online learning (NA, Slow, Fast, Average, No Internet access) 35. Average hours per day of yourself/child online learning during the MCO period (NA, <1, 1–3, 3–5, 5–8, >8) 36. Accessibility of faster Internet speed during online learning (NA, Morning, Night, Afternoon)

### Data preparation

There were 28 questions available for further analysis in this study, eliminating the missing ones. Question 32 and 33 were excluded from the survey because they focused on the primary food and non-food products purchased during the time of movement control orders. Given that the dataset was cluttered with missing values and errors, a considerable effort was spent on its cleaning before applying descriptive analytics techniques. Q34, Q35 and Q36 had missing values of 2071, 2243 and 2310 respectively and were replaced by the most frequent values. By cleaning the data, the raw data is transformed into structured data. Without losing any information, all lengthy responses were reduced to short and detailed responses. If the original answer for respondent’s dwelling state was "Wilayah Persekutuan Kuala Lumpur," it was converted to "KL." Questions with answers were labelled "Yes," whereas those without answers are labelled "No." One question, for example, inquired about respondents' willingness to eat out as part of the new norm's lifestyle adjustments. Those who agreed said they "will not eat out." Those who opposed to the shift in lifestyle left the question unanswered. As a result, these questions were changed to a "Yes" or "No" format.

### Modelling

Various machine learning techniques were used in this phase to meet the study objectives. To create prediction models, we used four machine learning techniques: Decision Tree, Random Forest, Gradient Boosted Tree and Naïve Bayes. These four machine learning techniques were chosen from a literature review ( Mostafa *et al.*, 2021; Sangavi *et al.*, 2020) and used to determine the optimal model by adjusting their parameters. Feature selection methods, such as Gini index, Gain–Ratio and various partitioning ratios of the training and test data sets were also compared ( Trivedi, 2020).

### Evaluation

In this phase, the best predictive model for each subsidy was selected based on the standard performance evaluation metrics: Sensitivity, Precision, F-Score and Accuracy ( Moscato *et al.*, 2021). The formulas used to calculate each of the metrics are given below.

$$Accuracy\;=\;\frac{TP\;+\;TN}{TP\;+\;TN\;+\;FP\;+\;FN}$$

TP = True Positive, TN = True Negative, FP = False Positive, FN = False Negative

The completeness of a prediction model was measured by sensitivity, also known as recall and total positive rate (TPR). This metric determined the proportion of positive predictions by a model that corresponds to true positive values ( Moscato *et al.*, 2021). The formula is given below.

$$Sensitivity\;=\;\frac{TP}{TP\;+\;FN}$$

Precision in data analytics refers to a model’s ability to correctly forecast outcomes. In other words, precision is a true positive divided by a combination of true and false positives.

$$Precision\;=\;\frac{TP}{TP\;+\;FP}$$

F-Score, also known as F1 Score, is a balance of both precision and sensitivity. Hence, this study used F-Score to evaluate the machine learning models.

$$F\;Score\;=\;2\times \;\frac{Precision\;\times \;Sensitivity}{Precision\;+\;Sensitivity}$$

### Deployment

In the final phase a deployment strategy for the model was created and documented. The best predictive model as determined for each of the subsidies was to be recommended for further deployment. The entire CRISP-DM phases were carried out using the Konstanz Information Miner (KNIME 4.3.2), a free and open-source data analytics software.

### Ethical approval

This study was carried out from November 23, 2020 to October 06, 2021 and has obtained ethics approval (EA1322021) by Technology Transfer Office, secretariat of research ethics committee, Multimedia university.

## Results

The outcomes of this study were organised as descriptive analytics, model optimisation and findings. Descriptive analytics helps to understand the characteristics of each respondent and the relationship between variables. Table 2 provides the descriptive information on the respondents.

**Table 2.** Descriptive statistics of respondent demographics.

State		Age Group	
Selangor Johor Sabah KL Perak Kedah Pahang Melaka Sarawak Negeri Sembilan Kelantan Pulau Pinang Terengganu Putrajaya Perlis Labuan	27.74% 12.35% 8.41% 7.84% 6.29% 4.96% 4.87% 4.83% 4.52% 4.38% 3.30% 3.23% 3.02% 2.94% 0.83% 0.50%	35–44 25–34 45–54 15–24 55–64 65 and above	38.87% 29.19% 19.38% 6.6% 5.46% 0.50%
		Marital Status	
		Married Single/ Non-Married Divorced/ Separated Single mother Widow/ Widower	70.95% 24.35% 1.98% 1.42% 1.28%
		Location	
		City Rural	70.71% 29.28%
Gender		Number of dependents including self	
Female Male	55.7% 44.3%	3–4 5–10 1–2 More than 10 people	36.21% 35.2% 28.1% 0.47%
Ethnic Group		Job status	
Malay Native Sabah/ Sarawak Chinese Indian Others Foreigner	79.61% 10.29% 7.18% 1.75 1.07 0.1%	Government Servants Private Sector Employees Not Working GLC Employees Self-Employed Employers MNC Employees Unpaid family workers	39.22% 27.14% 21.61% 4.35% 3.05% 2.18% 2.12% 0.32%

**Figure 2.**
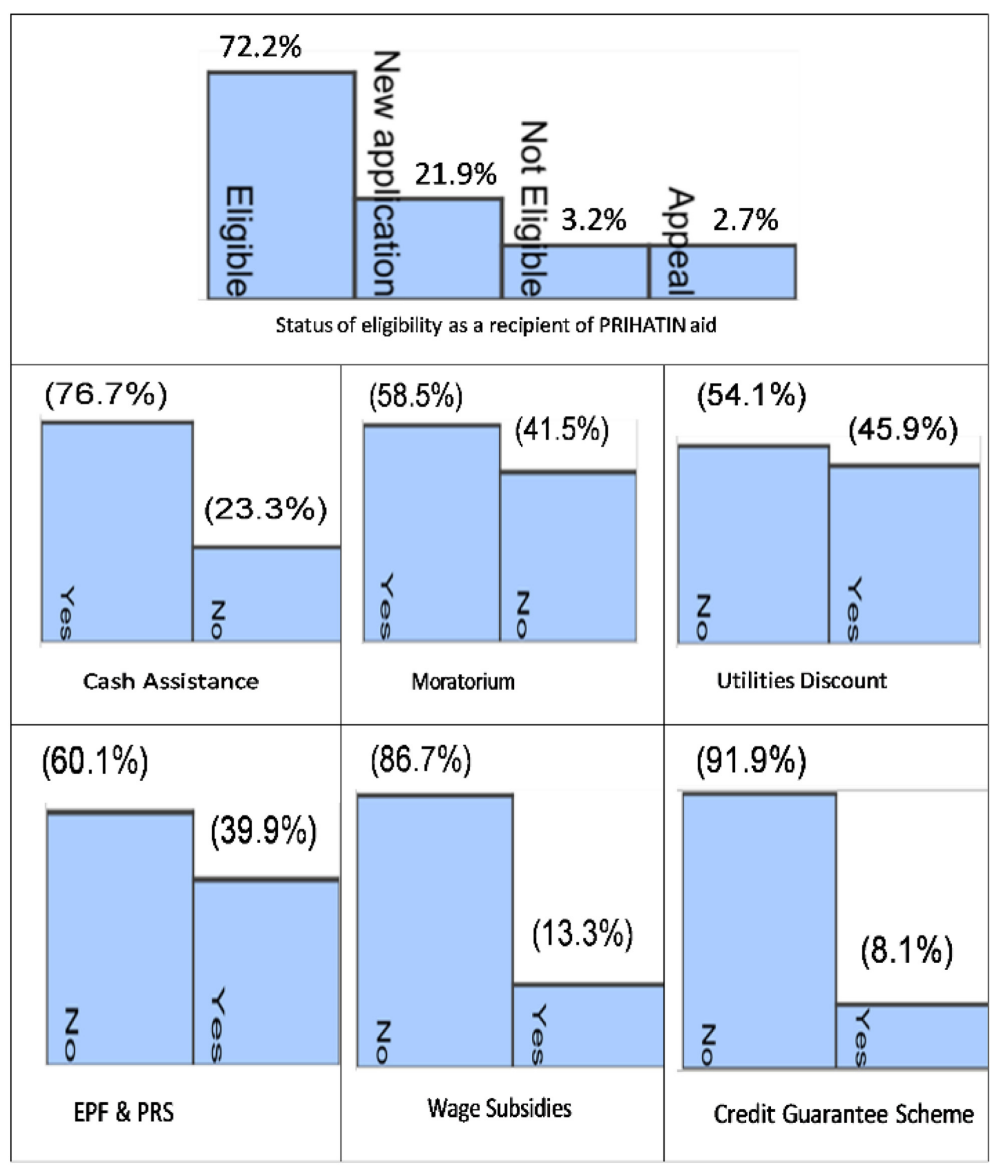
Most beneficial forms of assistance received under the Prihatin Rakyat ESP.

Figure 2 shows the various types of subsidies offered in the ESP. Among the 41,386 respondents, 72.2% were eligible to receive subsidies, 21.9% were newly applied, 3.2% were not eligible and 2.7% had appealed. Figure 2 also shows the most beneficial forms of support. The most popular type of subsidy was cash allowance, followed by moratorium, utility discounts and EPF and PRS cash withdrawals. The least preferred type was the credit guarantee plan and wage subsidies.

Following the descriptive analytics, the four machine learning techniques were applied to develop prediction models for each moratorium, utility discount and EPF withdrawals subsidies. Decision Tree, Gradient Boosted Tree, Random Forest and Naïve Bayes are subjected to parameter tuning to determine the best model and parameter values.

Table 3 to Table 6 show how the optimal model was obtained from each machine learning technique. Partitioning ratio indicates the training and test data. Gain ratio, Gini index and information gain were used to measure the quality of each predictor in classifying the target variable. The results show that the Gradient Boosted Tree and Naïve Bayes techniques performed well when 60% of the data were used to train the machine learning models and the other 40% was used for testing. Random Forest and Decision Tree techniques generated the best models when the training data were 80% and the test data were 20%. F-Score was used as the evaluation measure to select the optimal models. After identifying the optimal models, the best was selected among the four machine learning techniques. Table 7 shows the results. Gradient Boosted Tree outperformed the rest of the techniques in predicting the moratorium preference with 93.8% sensitivity, 82.1% precision and 87.6% F-score.

**Table 3.** Gradient Boosted Tree - Parameter tuning and identification of optimal model.

Machine Learning Technique	Partitioning Ratio	Target Variable: Moratorium	Precision	Sensitivity	F-score	Accuracy
Gradient Boosted Tree	50:50	No	0.877	0.71	0.784	0.838
		Yes	0.819	0.929	0.871	
	60:40	No	0.891	0.711	**0.791**	**0.844**
		Yes	0.821	**0.938**	**0.876**	
	70:30	No	0.883	0.717	0.791	0.843
		Yes	0.823	0.933	0.874	
	80:20	No	0.881	0.701	0.781	0.837
		Yes	0.815	0.933	0.87	

**Table 4.** Naïve Bayes - Parameter tuning and identification of optimal model.

Machine Learning Technique	Partitioning Ratio	Target Variable: Moratorium	Precision	Sensitivity	F-score	Accuracy
Naïve Bayes	50:50	No	0.627	0.449	0.524	0.661
		Yes	0.675	0.811	0.737	
	60:40	No	0.64	0.453	**0.53**	0.667
		Yes	0.679	0.819	**0.742**	
	70:30	No	0.633	0.449	0.526	0.663
		Yes	0.676	0.815	0.739	
	80:20	No	0.624	0.443	0.518	0.658
		Yes	0.673	0.811	0.735	

**Table 5.** Decision Tree - Parameter tuning and identification of optimal model.

Machine Learning Technique	Partitioning Ratio	Target Variable: Moratorium	Precision	Sensitivity	F-score	Accuracy
Decision Tree	50:50 (Gini Index)	No	0.776	0.705	0.739	0.793
		Yes	0.804	0.856	0.829	
	50:50 (Gain Ratio)	No	0.745	0.685	0.731	0.772
		Yes	0.789	0.834	0.811	
	60:40 (Gini Index)	No	0.772	0.697	0.733	0.789
		Yes	0.799	0.855	0.826	
	60:40 (Gain Ratio)	No	0.746	0.669	0.705	0.768
		Yes	0.781	0.839	0.809	
	70:30 (Gini Index)	No	0.766	0.7	0.731	0.787
		Yes	0.8	0.848	0.823	
	70:30 (Gain Ratio)	No	0.75	0.685	0.716	0.775
		Yes	0.79	0.838	0.813	
	80:20 (Gini Index)	No	0.779	0.727	**0.752**	**0.802**
		Yes	0.816	0.854	**0.834**	
	80:20 (Gain Ratio)	No	0.743	0.697	0.719	0.774
		Yes	0.794	0.83	0.812	

**Table 6.** Random Forest - Parameter tuning and identification of optimal model.

Machine Learning Technique	Partitioning Ratio	Target Variable: Moratorium	Precision	Sensitivity	F-score	Accuracy
Random Forest	50:50 (Information Gain)	No	0.809	0.766	0.787	0.828
		Yes	0.84	0.872	0.856	
	50:50 (Gain Ratio)	No	0.805	0.744	0.773	0.819
		Yes	0.828	0.872	0.849	
	50:50 (Gini Index)	No	0.804	0.763	0.783	0.825
		Yes	0.838	0.868	0.853	
	60:40 (Information Gain)	No	0.809	0.752	0.779	0.823
		Yes	0.833	0.874	0.853	
	60:40 (Gain Ratio)	No	0.804	0.739	0.77	0.817
		Yes	0.825	0.873	0.848	
	60:40 (Gini Index)	No	0.803	0.759	0.78	0.823
		Yes	0.836	0.868	0.852	
	70:30 (Information Gain)	No	0.808	0.774	0.791	0.83
		Yes	0.845	0.87	0.857	
	70:30 (Gain Ratio)	No	0.821	0.753	0.786	0.83
		Yes	0.835	0.884	0.859	
	70:30 (Gini Index)	No	0.815	0.765	0.789	0.831
		Yes	0.84	0.877	0.858	
	80:20 (Information Gain)	No	0.813	0.759	0.785	0.827
		Yes	0.837	0.876	0.856	
	80:20 (Gain Ratio)	No	0.825	0.752	0.787	0.831
		Yes	0.835	0.887	0.86	
	80:20 (Gini Index)	No	0.825	0.769	**0.796**	0.837
		Yes	0.844	0.885	**0.864**	

**Table 7.** Evaluation of predictive models: moratorium.

Machine Learning Technique	Partitioning Ratio	Target Variable: *Moratorium*	Precision	Sensitivity	F-score	Accuracy
Decision Tree	80:20(Gini Index)	No	0.779	0.727	0.752	0.802
		Yes	0.816	0.854	0.834	
Gradient Boosted Tree	60:40	No	0.891	0.711	0.791	0.844
		Yes	0.821	0.938	0.876	
Random Forest	80:20(Gini Index)	No	0.825	0.769	0.796	0.837
		Yes	0.844	0.885	0.864	
Naïve Bayes	60:40	No	0.64	0.453	0.53	0.667
		Yes	0.679	0.819	0.742	

A similar process was carried out to develop machine learning models for utility discounts and EPF and PRS subsidies. The results show that for both subsidies, Gradient Boosted Tree was the best machine learning technique. Table 8 and Table 9 show that this technique can predict utility discount with 86% sensitivity, 82.1% precision and 84% F-score, as well as EPF and PRS with 83.6% sensitivity, 81.2% precision and 82.4% F-score, respectively.

**Table 8.** Evaluation of predictive models: utilities discount.

Machine Learning Technique	Partitioning Ratio	Target Variable: *Utilities Discount*	Precision	Sensitivity	F-score	Accuracy
Decision Tree	80:20 (Gini Index)	No	0.849	0.835	0.842	0.83
		Yes	0.809	0.824	0.816	
Gradient Boosted Tree	80:20	No	0.876	0.841	0.859	0.85
		Yes	0.821	0.86	0.84	
Random Forest	80:20 (Information Gain Ratio)	No	0.848	0.864	0.856	0.843
		Yes	0.836	0.817	0.827	
Naïve Bayes	60:40	No	0.643	0.623	0.633	0.609
		Yes	0.571	0.592	0.581	

**Table 9.** Evaluation of predictive models: EPF and PRS withdrawals.

Machine Learning Technique	Partitioning Ratio	Target Variable: *EPF & PRS*	Precision	Sensitivity	F-score	Accuracy
Decision Tree	80:20 (Gini Index)	No	0.882	0.854	0.868	0.844
		Yes	0.791	0.828	0.809	
Gradient Boosted Tree	60:40	No	0.889	0.871	0.88	0.857
		Yes	0.812	0.836	0.824	
Random Forest	60:40 (Information Gain Ratio)	No	0.87	0.892	0.881	0.855
		Yes	0.831	0.799	0.815	
Naïve Bayes	60:40	No	0.699	0.781	0.738	0.666
		Yes	0.599	0.494	0.541	

## Discussion

In this study, Gradient Boosted Tree was found as the best machine learning model for predicting moratorium subsidies. However, this model cannot explain the relationship between the predictors and the target, and thus decision tree rules were derived to understand the overall profile of households who prefer a moratorium. Rule support refers to the number of respondents to whom this condition applies. Rule confidence indicates the probability of having a moratorium as the preferred subsidy. Table 10 shows the basic characteristics of families that choose moratorium subsidies with a rule support of 400 and above.

**Table 10.** General profiles for moratorium subsidy preference.

Condition	Rule Support	Rule Confidence
$Q12: Cash Assistance$ IN ("Yes") AND $Q12: Utilities Discount$ IN ("No") AND $Q12: EPF & PRS$ IN ("No") AND $Q12: Wage Sub$ IN ("Yes") AND $Q12: CGS$ IN ("No") AND $Q10: Race$ IN ("Malay", "Native Sabah/Sarawak", "Others") AND $Q7: Age Group$ IN ("35-44 ", "25-34 ", "45-54 ", "55-64 ")	902	95.68%
$Q22: Outbreak lifestyle changes?$ IN ("Yes") AND $Q4: Gender$ IN ("Male") AND $Q34: Internet access lvl$ IN ("NA", "Fast") AND $Q7: Age Group$ IN ("35-44 ") AND $Q5: Area$ IN ("City") AND $Q7: Age Group$ IN ("35-44 ", "25-34 ", "45-54 ") AND $Q8: Marital Status$ IN ("Married") AND $Q12: Wage Sub$ IN ("No") AND $Q12: CGS$ IN ("No") AND $Q10: Race$ IN ("Malay", "Native Sabah/Sarawak", "Others") AND $Q7: Age Group$ IN ("35-44 ", "25-34 ", "45-54 ", "55-64 ")	792	88.51%
$Q23: Readiness of lifestyle changes$ IN ("Yes") AND $Q12: EPF & PRS$ IN ("Yes") AND $Q12: Utilities Discount$ IN ("No") AND $Q22: Outbreak lifestyle changes?$ IN ("Yes") AND $Q2: State$ IN ("Selangor", "Perak", "Sarawak", "Kedah", "KL", "Johor", "Perlis", "Putrajaya", "Pahang", "Melaka", "Pulau Pinang", "Terengganu", "Negeri Sembilan", "Sabah", "Labuan") AND $Q4: Gender$ IN ("Male") AND $Q18: Future ESP?$ IN ("Yes") AND $Q12: Cash Assistance$ IN ("Yes") AND $Q5: Area$ IN ("Rural") AND $Q7: Age Group$ IN ("35-44 ", "25-34 ", "45-54 ") AND $Q8: Marital Status$ IN ("Married") AND $Q12: Wage Sub$ IN ("No") AND $Q12: CGS$ IN ("No") AND $Q10: Race$ IN ("Malay", "Native Sabah/Sarawak", "Others") AND $Q7: Age Group$ IN ("35-44 ", "25-34 ", "45-54 ", "55-64 ")	466	93.13%
$Q12: EPF & PRS$ IN ("Yes") AND $Q12: Utilities Discount$ IN ("No") AND $Q12: Cash Assistance$ IN ("Yes") AND $Q34: Internet access lvl$ IN ("NA", "Slow", "Average", "No Internet Access") AND $Q22: Outbreak lifestyle changes?$ IN ("Yes") AND $Q4: Gender$ IN ("Female") AND $Q2: State$ IN ("Selangor", "Kedah", "KL", "Johor", "Perlis", "Putrajaya", "Pulau Pinang", "Negeri Sembilan", "Sabah") AND $Q7: Age Group$ IN ("25-34 ", "45-54 ", "15-24 ", "55-64 ", "65 and above") AND $Q5: Area$ IN ("City") AND $Q7: Age Group$ IN ("35-44 ", "25-34 ", "45-54 ") AND $Q8: Marital Status$ IN ("Married") AND $Q12: Wage Sub$ IN ("No") AND $Q12: CGS$ IN ("No") AND $Q10: Race$ IN ("Malay", "Native Sabah/Sarawak", "Others") AND $Q7: Age Group$ IN ("35-44 ", "25-34 ", "45-54 ", "55-64 ")	447	90.16%

The first rule shows that households who prefer to have a cash allowance and their race is either Malay or Native Sabah/Sarawak or others, while those aged between 25 to 64 prefer moratorium. Table 11 explains the first rule indicating the general profile of households who prefer moratorium subsidies. Similarly, Table 12 and Table 13 show the general profile of households preferring utility discounts and EPF and PRS cash withdrawals.

**Table 11.** General profile of moratorium subsidy preference - rule support: 902 records, rule confidence: 95.7%.

Subsidies	Race	Age Group
• Cash Assistance (Yes) • Utilities Discount (No) • EOF & PRS (No) • Wage Subsidies (No) • CGS (No)	• Malay • Native Sabah / Sarawak • Others	• 35-44 • 25-34 • 45-54 • 55-64

**Table 12.** General profile of utilities discount subsidy preference - rule support: 1128 records, rule confidence: 90.1%.

Readiness of Lifestyle Changes	Marital Status	States	Subsidies
Yes	• Married • Divorced / Separated	• KL • Putrajaya • Melaka • Pulau Penang • Sarawak • Johor	• CGS (No) • Cash Assistance (Yes) • Wage Subsidies (No) • Moratorium (Yes) • EPF & PRS (No)

**Table 13.** General profile of EPF & PRS subsidy preference - rule support: 1427 records, rule confidence: 90.5%.

ESP in future	Marital status	Priority	Race	States	Subsides
Yes	• Single / Non-Married • Married • Widow / Widower • Divorced / Separated	Employment	• Malay • Native Sabah / Sarawak • Others • Foreigner	• KL • Selangor • Johor • Labuan • Sarawak • Negeri Sembilan	• CGS (No) • Cash Assistance (Yes) • Wage Subsidies (No) • Moratorium (Yes) • Utilities Discount (No)

The results imply that households that prefer moratorium subsidies did not favour other financial aids except cash assistance. By contrast, households that prefer for utility discounts, EPF and PRS withdrawals also chose moratorium subsidies and cash assistance. All households preferred cash assistance, which had the highest score among financial aids, followed by moratorium subsidies. Utility discounts, EPF and PRS withdrawals can be implemented according to the household income group preferences. 

Wage subsidy and credit guarantee scheme were the least preferred financial assistance. First, the Prihatin wage subsidy is only for eligible Social Security Organisation (SOCSO) subscribers. Hawkers, small businesses and their employees might not subscribe to SOCSO and thus, ineligible to apply for financial aid. Second, the credit guarantee scheme was not preferred due to economic uncertainty from COVID-19. Economic uncertainty adversely affects household income, resulting in their inability to repay the loan instalments. 

### Limitations of the study

The following are some of the limitations of this study's findings: The data used are survey responses and cannot be considered to represent the views of all Malaysians. According to DOSM, it should not be used to analyse the impact of COVID-19 in Malaysia and should not be considered official statistics. It can, however, be utilised to assist in the reflection process ( “Department of Statistics Malaysia Official Portal,” 2020; Malaysia, 2020).

## Conclusions

This study used data analytics and machine learning approaches to derive insights from the “Effects of COVID-19 on the Economy and Individual - Round 2” survey dataset. The CRISP-DM approach was applied to develop prediction models for households’ preferred subsidies, such as moratoriums, utility discounts and EPF and PRS using four machine learning algorithms, namely, Decision Tree, Random Forest, Naïve Bayes and Gradient Boosted Tree. For all three subsidies, the best predictive model was obtained by Gradient Boosted Tree. The findings can be used to design customised ESPs that effectively manage the economic burden of low-income households.

## Data availability

Data used in this study were obtained from a survey dataset “Effects of COVID-19 on the Economy and Individual - Round 2,” available from the Department of Statistics, Malaysia (DOSM). A report published by the DOSM based on the survey can be viewed on the DOSM website. Access to this data requires application, as stated on the DOSM website. A guide for how to apply for dataset access is available on the Data Request page or requests for more information can be emailed to data@dosm.gov.my.
