# Supplementary material for: COVID-19 impact: Customised economic stimulus package recommender system using machine learning techniques
Source: F1000Res. 2021 Nov 12;10:932. Originally published 2021 Sep 16. [Version 2] doi: 10.12688/f1000research.72976.2 (PMC8647044; doi:10.12688/f1000research.72976.2)
Supplement: Peer review history

# Reviewer report: Reviewer response for version 2

**DOI:** 10.5256/f1000research.79204.r100081

**Published:** 2024-05-31

**Authors:** Setia Pramana

**Affiliations:** 1 BPS Statistics Indonesia, Jakarta, Indonesia; 2 Computational Statistics Department, Politeknik, Statistika STIS, Jakarta, Indonesia

The authors have addressed all suggested comments. I have no further comments to make.

Is the work clearly and accurately presented and does it cite the current literature?

Partly

If applicable, is the statistical analysis and its interpretation appropriate?

Partly

Are all the source data underlying the results available to ensure full reproducibility?

Yes

Is the study design appropriate and is the work technically sound?

Yes

Are the conclusions drawn adequately supported by the results?

Yes

Are sufficient details of methods and analysis provided to allow replication by others?

Partly

Reviewer Expertise:

Machine Learning, Data mining, statistics, official statistics, bioinformatics, biostatistics

I confirm that I have read this submission and believe that I have an appropriate level of expertise to confirm that it is of an acceptable scientific standard.

# Reviewer report: Reviewer response for version 2

**DOI:** 10.5256/f1000research.79204.r100079

**Published:** 2021-12-03

**Authors:** Sreeja N.K

**Affiliations:** 1 Department of Applied Mathematics and Computational Sciences, PSG College of Technology, Coimbatore, Tamil Nadu, India

The authors have addressed the issues raised.

 The authors have reported the average results of cross validation.

Is the work clearly and accurately presented and does it cite the current literature?

Yes

If applicable, is the statistical analysis and its interpretation appropriate?

No

Are all the source data underlying the results available to ensure full reproducibility?

Yes

Is the study design appropriate and is the work technically sound?

Yes

Are the conclusions drawn adequately supported by the results?

Yes

Are sufficient details of methods and analysis provided to allow replication by others?

Yes

Reviewer Expertise:

Pattern recognition, Supervised Learning, Swarm Intelligence

I confirm that I have read this submission and believe that I have an appropriate level of expertise to confirm that it is of an acceptable scientific standard.

# Reviewer report: Reviewer response for version 1

**DOI:** 10.5256/f1000research.76592.r97108

**Published:** 2021-10-25

**Authors:** Sreeja N.K

**Affiliations:** 1 Department of Applied Mathematics and Computational Sciences, PSG College of Technology, Coimbatore, Tamil Nadu, India

- The authors have compared few classifiers on Effects of COVID-19 on the Economy and Individual - Round 2 data set to predict individual household preferences from ESP.
- I would recommend the authors to use k-fold cross validation method to evaluate the performance of a classifier instead of a random 60-40 or 80-20 split. This would guarantee that all k subsets are used as a test set once. 
- The authors can also perform some statistical tests to validate the results obtained from experiments.
- In Page 4, (section Evaluation), TPR is the True positive rate and not as mentioned by the authors.

Is the work clearly and accurately presented and does it cite the current literature?

Yes

If applicable, is the statistical analysis and its interpretation appropriate?

No

Are all the source data underlying the results available to ensure full reproducibility?

Yes

Is the study design appropriate and is the work technically sound?

Yes

Are the conclusions drawn adequately supported by the results?

Yes

Are sufficient details of methods and analysis provided to allow replication by others?

Yes

Reviewer Expertise:

Supervised Learning, Swarm Intelligence

I confirm that I have read this submission and believe that I have an appropriate level of expertise to confirm that it is of an acceptable scientific standard, however I have significant reservations, as outlined above.

# Response

**Published:** 2021-11-12

**Authors:** Rathimala Kannan

**Affiliations:** Multimedia University, Malaysia

- I would recommend the authors to use k-fold cross validation method to evaluate the performance of a classifier instead of a random 60-40 or 80-20 split. This would guarantee that all k subsets are used as a test set once. 

**Thank you for your comments.  As we are aimed to tune the parameters to get the optimal model from each machine learning techniques, separate tables were provided with the various parameter values. Also the data size was 40k.**

**We conducted K-fold cross validation method to evaluate the performance of a classifier. When the data is partitioned with k= 5, and K=10, the findings reveal little difference in F-score values, and the Gradient boosting tree performs best. When the data set is small, k-fold cross validation produces a superior model; but, when the data set is large, it produces no change. A current study backs up this conclusion (Marcot & Hanea, 2021).**

 Marcot, B. G., & Hanea, A. M. (2021). What is an optimal value of k in k-fold cross-validation in discrete Bayesian network analysis? *Computational Statistics*, *36*(3), 2009–2031. https://doi.org/10.1007/s00180-020-00999-9

**Machine Learning Technique**

**Partitioning Ratio**

**Target Variable:** ***Moratorium***

**Precision**

**Sensitivity**

**F-score**

**Accuracy**

 Decision Tree

 80:20   

 (Gini Index)

 No

 0.779

 0.727

 0.752

 0.802

 Yes

 0.816

 0.854

 0.834

 K=5-fold cross validation

 (Gini Index)

 No

 0.770

 0.702

 0.735

 0.789

 Yes

 0.801

 0.851

 0.826

 K=10-fold cross validation

 (Gini Index)

 No

 0.772

 0.697

 0.733

 0.789

 Yes

 0.799

 0.854

 0.826

 Gradient Boosted Tree

 60:40

 No

 0.891

 0.711

 0.791

 0.844

 Yes

 0.821

 0.938

 0.876

 K=5-fold cross validation

 No

 0.889

 0.705

 0.786

 0.841

 Yes

 0.818

 0.938

 0.874

 K=10-fold cross validation

 No

 0.889

 0.705

 0.787

 0.841

 Yes

 0.818

 0.938

 0.874

 Random Forest

 80:20   

 (Gini Index)

 No

 0.825

 0.769

 0.796

 0.837

 Yes

 0.844

 0.885

 0.864

 K=5-fold cross validation

 (Gini Index)

 No

 0.821

 0.761

 0.790

 0.832

 Yes

 0.839

 0.882

 0.860

 K=10-fold cross validation

 (Gini Index)

 No

 0.826

 0.757

 0.790

 0.833

 Yes

 0.837

 0.887

 0.862

 Naïve Bayes

 60:40

 No

 0.64

 0.453

 0.53

 0.667

 Yes

 0.679

 0.819

 0.742

 K=5-fold cross validation

 No

 0.634

 0.446

 0.524

 0.664

 Yes

 0.676

 0.818

 0.740

 K=10-fold cross validation

 No

 0.634

 0.446

 0.524

 0.664

 Yes

 0.676

 0.818

 0.740

  

- In Page 4, (section Evaluation), TPR is the True positive rate and not as mentioned by the authors.

**Thank you for spotting this typo error. We have corrected it.**

# Reviewer report: Reviewer response for version 1

**DOI:** 10.5256/f1000research.76592.r95431

**Published:** 2021-10-15

**Authors:** Shahrinaz Ismail

**Affiliations:** 1 Albukhary International University, Alor Setar, Kedah, Malaysia

Since this is a full research paper, it is recommended that a Literature Review section be included. It is believed that there are some previous research that have performed the 4 ML techniques covered by this research, hence the need to also mention those and how they differ in results as compared to the results found in here.

 Some additional elaboration is required for technical parts of this paper, especially in clarifying the relation between the formula and the techniques used. Do all the formulas need to be performed for each of the 4 techniques?

 If there is a step taken before the evaluation that uses the formulas, please include them at least as summary. If this part is covered in the published paper (part 1), perhaps that should be mentioned. If this paper only covers the result on which technique is the best, then perhaps the title should reflect that. At the moment, the title does not reflect the research questions.

 Research questions are usually asked with the word "how" instead of "can". It would be more sound to rephrase them to "how".

Is the work clearly and accurately presented and does it cite the current literature?

Yes

If applicable, is the statistical analysis and its interpretation appropriate?

Yes

Are all the source data underlying the results available to ensure full reproducibility?

Partly

Is the study design appropriate and is the work technically sound?

Yes

Are the conclusions drawn adequately supported by the results?

Partly

Are sufficient details of methods and analysis provided to allow replication by others?

Yes

Reviewer Expertise:

Data analytics with knowledge management

I confirm that I have read this submission and believe that I have an appropriate level of expertise to confirm that it is of an acceptable scientific standard, however I have significant reservations, as outlined above.

# Response

**Published:** 2021-11-12

**Authors:** Rathimala Kannan

**Affiliations:** Multimedia University, Malaysia

Since this is a full research paper, it is recommended that a Literature Review section be included. It is believed that there are some previous research that have performed the 4 ML techniques covered by this research, hence the need to also mention those and how they differ in results as compared to the results found in here.

**We have added brief literature review in the modelling section. Due to the limitation of total words to be within 2500 words, a separate section is not added.**

 Some additional elaboration is required for technical parts of this paper, especially in clarifying the relation between the formula and the techniques used. Do all the formulas need to be performed for each of the 4 techniques?

**We employed standard performance indicators to evaluate the classification models.**

 If there is a step taken before the evaluation that uses the formulas, please include them at least as summary. If this part is covered in the published paper (part 1), perhaps that should be mentioned. If this paper only covers the result on which technique is the best, then perhaps the title should reflect that. At the moment, the title does not reflect the research questions.

**We have revised the discussion section which summarizes the process and the results.**

 Research questions are usually asked with the word "how" instead of "can". It would be more sound to rephrase them to "how".

**The research questions are rephrased to start with "how".**

# Reviewer report: Reviewer response for version 1

**DOI:** 10.5256/f1000research.76592.r95430

**Published:** 2021-10-06

**Authors:** Setia Pramana

**Affiliations:** 1 BPS Statistics Indonesia, Jakarta, Indonesia; 2 Computational Statistics Department, Politeknik, Statistika STIS, Jakarta, Indonesia

1. Research questions and the discussion and conclusion are not inline. The authors mentioned three research questions, but they are not discussed throughout the manuscript. Instead, the authors focus on comparing different methods for predicting household subsidy preferences. Please address the research question on the results and discussion as well.
2. Please explain the question used in the study and why some questions are missing. Is it due to not being the focus of the study, or for other reasons?
3. Please elaborate more on the discussion of the results of the study. What would be the impact of utilizing best methods for result of the stimulus, especially for the citizen? Would it be that big a difference if we use the other classification methods?
4. Please explain the advantages and disadvantages of the methods being compared in the study.
5. The result shows that the decision tree, gradient boost, and random forest, does not differ much, how will it affect the stimulus package?
6. If the data changes would the results change? The authors may divide the data into testing, training and validation data set. Several times as discussed by this paper: Lessman *et al.* (2015 ^1^).
7. Please use a flowchart to explain the modelling and analysis process.
8. Why discuss on different ratio partitioning, in this case it is better use simulation dataset to see the impact of the ratio into the results.

Is the work clearly and accurately presented and does it cite the current literature?

Partly

If applicable, is the statistical analysis and its interpretation appropriate?

Partly

Are all the source data underlying the results available to ensure full reproducibility?

Yes

Is the study design appropriate and is the work technically sound?

Yes

Are the conclusions drawn adequately supported by the results?

Yes

Are sufficient details of methods and analysis provided to allow replication by others?

Partly

Reviewer Expertise:

Machine Learning, Data mining, statistics, official statistics, bioinformatics, biostatistics

I confirm that I have read this submission and believe that I have an appropriate level of expertise to confirm that it is of an acceptable scientific standard, however I have significant reservations, as outlined above.

## References

[1] : Benchmarking state-of-the-art classification algorithms for credit scoring: An update of research. *European Journal of Operational Research* .2015;247(1) : 10.1016/j.ejor.2015.05.030 124-136 10.1016/j.ejor.2015.05.030

# Response

**Published:** 2021-11-12

**Authors:** Rathimala Kannan

**Affiliations:** Multimedia University, Malaysia

1. Research questions and the discussion and conclusion are not inline. The authors mentioned three research questions, but they are not discussed throughout the manuscript. Instead, the authors focus on comparing different methods for predicting household subsidy preferences. Please address the research question on the results and discussion as well.

**Though we have answered all three research questions in the discussion section, we noticed it is not presented well. Thank you for  your comment. Now we have answered each question separately in the discussion section.**

 2. Please explain the question used in the study and why some questions are missing. Is it due to not being the focus of the study, or for other reasons?

**As mentioned in the previous answer, now we have revised the discussion section which clearly addresses all three research questions**.

 3. Please elaborate more on the discussion of the results of the study. What would be the impact of utilizing best methods for result of the stimulus, especially for the citizen? Would it be that big a difference if we use the other classification methods?

**The aim of this study is to identify the households that prefer to have a specific subsidy which is expected to lessen their financial burden during this pandemic. Therefore, we focus on the classification model which can predict more accurately the household’s choice.**

**It is possible to test with other classification methods, and they might have better accuracy. However, we focus also the model interpretability, thus we use mostly on decision tree algorithm family plus naïve bayes algorithm. Machine learning models are always a trade-off between accuracy and interpretability (Hastie, Tibshirani, James, & Witten, 2021).**

**Hastie, T., Tibshirani, R., James, G., & Witten, D. (2021). An Introduction to Statistical Learning (2nd Edition).** *Springer Texts* **,** *102* **, 618. (chapter 2.1.3 page 24)**

 4. Please explain the advantages and disadvantages of the methods being compared in the study.

**Different approaches, such as information-based learning, similarity-based learning, error-based learning, and probability-based learning, can be used to create classifiers. This research used information based learning (a decision tree algorithm family) as the best model for explaining decision logic .(Kelleher, Namee, & D’Arcy, 2015)**

**Kelleher, J. D., Namee, B. Mac, & D’Arcy, A. (2015). *Fundamentals of Machine Learning for Predictive Data Anayltics: Algorithms, Worked Examples, and Case Studies* (Ist). The MIT Press.**

 5. The result shows that the decision tree, gradient boost, and random forest, does not differ much, how will it affect the stimulus package?

**The aim here is to predict household’s preference as much possible correctly. Thus we chose the model that performs the best compared to the rest.**

 6. If the data changes would the results change? The authors may divide the data into testing, training and validation data set. Several times as discussed by this paper: Lessman *et al.* (2015 ^1^ ). –

**Yes. We can divide the dataset in to 3 sets. We conducted K-fold cross validation method to evaluate the performance of a classifier and presented the results in the following table. When the data is partitioned with k= 5, and K=10, the findings reveal little difference in classifier performance, and the Gradient boosting tree performs best. When the data set is small, k-fold cross validation produces a superior model; but, when the data set is large, it produces no change. A current study backs up this conclusion (Marcot & Hanea, 2021).**

**Marcot, B. G., & Hanea, A. M. (2021). What is an optimal value of k in k-fold cross-validation in discrete Bayesian network analysis? *Computational Statistics*, *36*(3), 2009–2031. https://doi.org/10.1007/s00180-020-00999-9**

**Machine Learning Technique**

**Partitioning Ratio**

**Target Variable:** ***Moratorium***

**Precision**

**Sensitivity**

**F-score**

**Accuracy**

 Decision Tree

 80:20   

 (Gini Index)

 No

 0.779

 0.727

 0.752

 0.802

 Yes

 0.816

 0.854

 0.834

 K=5-fold cross validation

 (Gini Index)

 No

 0.770

 0.702

 0.735

 0.789

 Yes

 0.801

 0.851

 0.826

 K=10-fold cross validation

 (Gini Index)

 No

 0.772

 0.697

 0.733

 0.789

 Yes

 0.799

 0.854

 0.826

 Gradient Boosted Tree

 60:40

 No

 0.891

 0.711

 0.791

 0.844

 Yes

 0.821

 0.938

 0.876

 K=5-fold cross validation

 No

 0.889

 0.705

 0.786

 0.841

 Yes

 0.818

 0.938

 0.874

 K=10-fold cross validation

 No

 0.889

 0.705

 0.787

 0.841

 Yes

 0.818

 0.938

 0.874

 Random Forest

 80:20   

 (Gini Index)

 No

 0.825

 0.769

 0.796

 0.837

 Yes

 0.844

 0.885

 0.864

 K=5-fold cross validation

 (Gini Index)

 No

 0.821

 0.761

 0.790

 0.832

 Yes

 0.839

 0.882

 0.860

 K=10-fold cross validation

 (Gini Index)

 No

 0.826

 0.757

 0.790

 0.833

 Yes

 0.837

 0.887

 0.862

 Naïve Bayes

 60:40

 No

 0.64

 0.453

 0.53

 0.667

 Yes

 0.679

 0.819

 0.742

 K=5-fold cross validation

 No

 0.634

 0.446

 0.524

 0.664

 Yes

 0.676

 0.818

 0.740

 K=10-fold cross validation

 No

 0.634

 0.446

 0.524

 0.664

 Yes

 0.676

 0.818

 0.740

 7. Please use a flowchart to explain the modelling and analysis process.

**A flowchart has been provided. Thank you for your comment.**

 8. Why discuss on different ratio partitioning, in this case it is better use simulation dataset to see the impact of the ratio into the results.

**Data partitioning ratio is considered as one of the parameters and the aim was to identify the best ratio that can provide better classification model.  We also used k-fold cross validation method as explained earlier.**
